# Association Between Social Frailty and Satisfaction With the Outcomes of Social Activities in Community‐Dwelling Older Adults in Japan: A Cross‐Sectional Study

**DOI:** 10.1111/psyg.70177

**Published:** 2026-05-06

**Authors:** Ryu Kobayashi, Yukihiro Gomi, Tomohiro Kakehi, Naotoshi Kimura, Hideaki Ishii, Takeki Ishida, Yoko Sakurai, Tomohiro Omori, Yusuke Nishida

**Affiliations:** ^1^ Department of Occupational Therapy International University of Health and Welfare Chiba Japan; ^2^ Department of Physical Therapy International University of Health and Welfare Chiba Japan; ^3^ Department of Speech Therapy International University of Health and Welfare Chiba Japan

**Keywords:** ageing, older adults, satisfaction, social activity, social frailty

## Abstract

**Background:**

Social frailty is characterised by a decline in social resources, engagement in social activities and self‐management capacity. Reduced subjective satisfaction with the outcomes of social activities is associated with social frailty. However, existing instruments do not adequately capture this dimension, and few studies have reported differences in satisfaction by severity of social frailty. The Social Activities‐Related Daily Life Satisfaction Scale (SARDLSS) provides a more comprehensive measure. We investigated the association between social frailty and satisfaction with the outcomes of social activities among community‐dwelling older adults in Japan.

**Methods:**

This cross‐sectional analysis included community‐dwelling adults aged ≥ 65 years who participated in a community health survey. Social frailty was assessed using the Makizako Social Frailty Index and stratified into robust, pre‐social frailty and social frailty. Satisfaction with the outcomes of social activities was quantified using the SARDLSS. Group differences were examined, and ordinal logistic regression models were fitted to estimate the association between social frailty status and satisfaction with the outcomes of social activities, adjusting for confounding factors, including age, walking speed and cognitive function.

**Results:**

A total of 141 participants were included in the analysis; 27.7% were robust, 36.9% had pre‐social frailty and 35.4% had social frailty. Ordinal logistic regression analyses indicated that higher satisfaction was independently associated with lower odds of belonging to a more severe social frailty category after adjusting for potential confounders (odds ratio = 0.95, 95% confidence interval: 0.91–0.98, *p* = 0.004). Domain‐level analyses corroborated this pattern, with satisfaction with friendships, health and physical fitness and contributions to others and society showing significant associations with social frailty status.

**Conclusion:**

Social frailty among community‐dwelling older adults was associated with their satisfaction with the outcomes of social activities. Addressing both quantitative and qualitative aspects of social engagement is warranted for preventing or mitigating social frailty.

## 1 Introduction

With global population ageing, increasing attention has been directed towards promoting healthy ageing, and frailty has emerged as a key concept. Frailty is a prevalent state of vulnerability in older adults and a multidimensional construct encompassing physical aspects as well as psychological and social domains [1]. Among these dimensions, social frailty has been conceptualised as a multidimensional condition that includes a decline in social resources essential for meeting basic social needs, reduced engagement in social behaviours and activities and diminished capacity for self‐management [2]. Social frailty has attracted growing international attention in recent years, with studies reporting an association with increased risks of cognitive decline, depressive symptoms, incident disability and mortality among older adults [3, 4, 5].

Risk factors for social frailty have been examined from multiple perspectives. Depressive symptoms, difficulties in activities of daily living (ADL), impaired physical function, cognitive decline and both excessive and insufficient sleep have been identified as risk factors that may contribute to the development and progression of social frailty [6, 7]. Social frailty has also been shown to be influenced by psychological factors, including subjective life satisfaction [8]. A study conducted by the authors using the Aid for Decision‐making in Occupation Choice (ADOC) to examine the association between satisfaction with meaningful activities and social frailty risk reported that lower subjective satisfaction with social activities, such as interpersonal interactions and leisure activities, was associated with social frailty [9]. These findings suggest that social frailty may be influenced by physical factors and by psychological factors, including individuals' subjective satisfaction with social activities.

However, the ADOC is designed to allow individuals to select activities that are meaningful to them and to rate their satisfaction with those activities [10]. Although this approach enables a high degree of individualisation, it may not capture satisfaction with social activities comprehensively and systematically. Moreover, previous studies have not sufficiently reported how subjective satisfaction with social activities varies by social frailty severity.

To address these limitations, the Social Activities‐Related Daily Life Satisfaction Scale (SARDLSS) developed by Okamoto offers a suitable instrument [11]. This scale is based on a comprehensive construct encompassing multiple dimensions of social activities, and its reliability and validity have been established. The use of this scale enables a systematic assessment of the degree to which individuals perceive that they derive meaningful benefits from social activities, and it is expected to facilitate a more detailed examination of its association with social frailty. Therefore, the aim of this study was to examine the association between social frailty and satisfaction with the outcomes of social activities among community‐dwelling older adults in Japan, focusing on differences across stages of social frailty.

## 2 Methods

### 2.1 Study Design and Participants

This cross‐sectional study was conducted using data from the community‐based health survey ‘Narita Kozu Study’, administered by the International University of Health and Welfare in 2025. The survey was conducted in collaboration with the local Community Comprehensive Support Center and the Social Welfare Council in the Kozu district of Narita City, Chiba Prefecture, Japan.

Participants were community‐dwelling older adults aged 65 years or older. Recruitment was conducted using flyers distributed through the local Social Welfare Council and the Community Comprehensive Support Center. The inclusion criterion was being a Japanese adult aged ≥ 65 years without long‐term care certification under the Japanese long‐term care insurance system. The exclusion criteria were as follows: (1) difficulty completing a self‐administered questionnaire owing to conditions such as dementia; (2) inability to understand the study purpose or provide informed consent and (3) missing primary data on social frailty and the SARDLSS.

Written informed consent was obtained from all participants prior to study participation. This study was approved by the Research Ethics Committee of the International University of Health and Welfare (approval number: 25‐CC‐016). All study procedures were conducted following the principles of the Declaration of Helsinki.

### 2.2 Measures

#### 2.2.1 Assessment of Social Frailty

Based on previous studies, social frailty was assessed using the Makizako Social Frailty Index (MSFI) [12]. The MSFI consists of the following five items: (1) decreased frequency of going out compared with the previous year (yes), (2) visiting friends' homes (no), (3) perceiving oneself as useful to family or friends (no), (4) living alone (yes) and (5) having daily conversations with someone (no). Participants who met two or more of these criteria were classified as having social frailty, those who met one criterion were classified as having pre‐social frailty, and those who met none were classified as robust. The construct validity of the MSFI has been established previously [13].

#### 2.2.2 Assessment of Satisfaction With the Outcomes of Social Activities

Satisfaction with the outcomes of social activities was assessed using the SARDLSS, developed by Okamoto [11]. This scale consists of 14 items across four domains: (1) satisfaction with learning‐related activities, (2) satisfaction with contributions to others and society, (3) satisfaction with health and physical fitness and (4) satisfaction with friendships. Each item is rated on a 5‐point Likert scale ranging from 1 (“not at all applicable”) to 5 (“very applicable”). The total score ranges from 14 to 70, with higher scores indicating greater satisfaction. The reliability and validity of the SARDLSS have been previously established [11]. In accordance with the original definition proposed by Okamoto [11], the SARDLSS assesses satisfaction with specific outcomes derived from participation in social activities. This construct reflects the degree to which older adults perceive their engagement in social activities as having generated meaningful benefits across four key domains: learning, contribution to others and society, health and physical strength and friendship. Notably, this concept is distinct from a general or global sense of satisfaction with social activities themselves.

The MSFI evaluates the behavioural and environmental aspects of social participation, including frequency of going out, social interactions, social roles and living arrangements. In contrast, the SARDLSS captures individuals' subjective evaluations of the outcomes resulting from such participation. Therefore, these two instruments assess fundamentally different dimensions of social participation and are treated as analytically independent constructs despite partial content similarity.

#### 2.2.3 Demographic Variables and Covariates

Demographic data, including age, sex, educational attainment, family composition and presence of chronic diseases, were collected. Functional status, cognitive function, depressive symptoms and ADL—all previously associated with social frailty—were assessed as covariates [6, 7]. Functional status was evaluated using usual 10‐m walking speed [14]. Cognitive function was assessed using the Japanese version of the Montreal Cognitive Assessment (MoCA‐J) [15]. Depressive symptoms were measured using the 15‐item Geriatric Depression Scale (GDS‐15) [16]. ADL was assessed using a self‐reported questionnaire covering five items: eating, grooming, bathing, walking and stair climbing. Participants who reported independence in all five items were classified as ADL‐independent.

### 2.3 Statistical Methods

Participants' demographic characteristics were summarised using descriptive statistics. Continuous variables were reported as means and standard deviations (SD) or medians and interquartile ranges (IQR), depending on data distribution. Categorical variables were presented as percentages.

Participants were classified into three groups based on their MSFI scores: social frailty, pre‐social frailty and robustness. One‐way analysis of variance (ANOVA) or the Kruskal–Wallis test was used to compare baseline characteristics and social activity satisfaction scores among the three groups. For categorical variables, the chi‐square test or Fisher's exact test was used, as appropriate. Post hoc comparisons were conducted when significant differences were identified.

Ordinal logistic regression was performed with social frailty status as the dependent variable and social activity satisfaction score as the primary independent variable. A basic model (Model 1) was first constructed, adjusting for age, sex and years of education. An extended model (Model 2) was then developed by additionally adjusting for walking speed, cognitive function and depressive symptoms.

Covariates were selected based on prior literature and theoretical considerations, including variables reported as correlates of social frailty, but not part of its construct, and were treated as potential confounders. Living arrangement (alone) was excluded from the regression models to avoid over‐adjustment, as it is a constituent item of the MSFI. In addition, ADL was excluded from the covariates because all participants were independent, resulting in no variability.

Age did not meet the proportional odds assumption when treated as a continuous variable; therefore, based on previous studies and the definition of late older age in Japan, age was dichotomised into two groups (< 75 and ≥ 75 years) [17, 18]. Correlations among independent variables were examined using Pearson's correlation coefficient or Spearman's rank correlation coefficient, and no substantial multicollinearity was observed. After confirming the proportional odds assumption, odds ratios (ORs) and 95% confidence intervals (CIs) were calculated.

As a secondary analysis, domain‐level analyses were conducted to examine the associations of four satisfaction domains— (1) learning‐related activities, (2) contributions to others and society, (3) health and physical fitness and (4) friendships—with social frailty status. Each domain score was analysed separately using ordinal logistic regression, adjusting for the same covariates as Model 2. To account for multiple comparisons across the four domains, Bonferroni correction was applied and the two‐sided significance level was set at *p* < 0.0125.

As a sensitivity analysis, the ordinal logistic regression models (Models 1 and 2) were additionally fitted after excluding the ‘satisfaction with contributions to others and society’ subdomain score from the SARDLSS total score, given its conceptual proximity to the MSFI item on perceiving oneself as useful to family or friends.

All statistical analyses were performed using IBM SPSS Statistics (version 29.0; IBM Corp., Armonk, NY, USA), and the level of statistical significance was set at *p* < 0.05.

## 3 Results

### 3.1 Participant Characteristics

**FIGURE 1:**
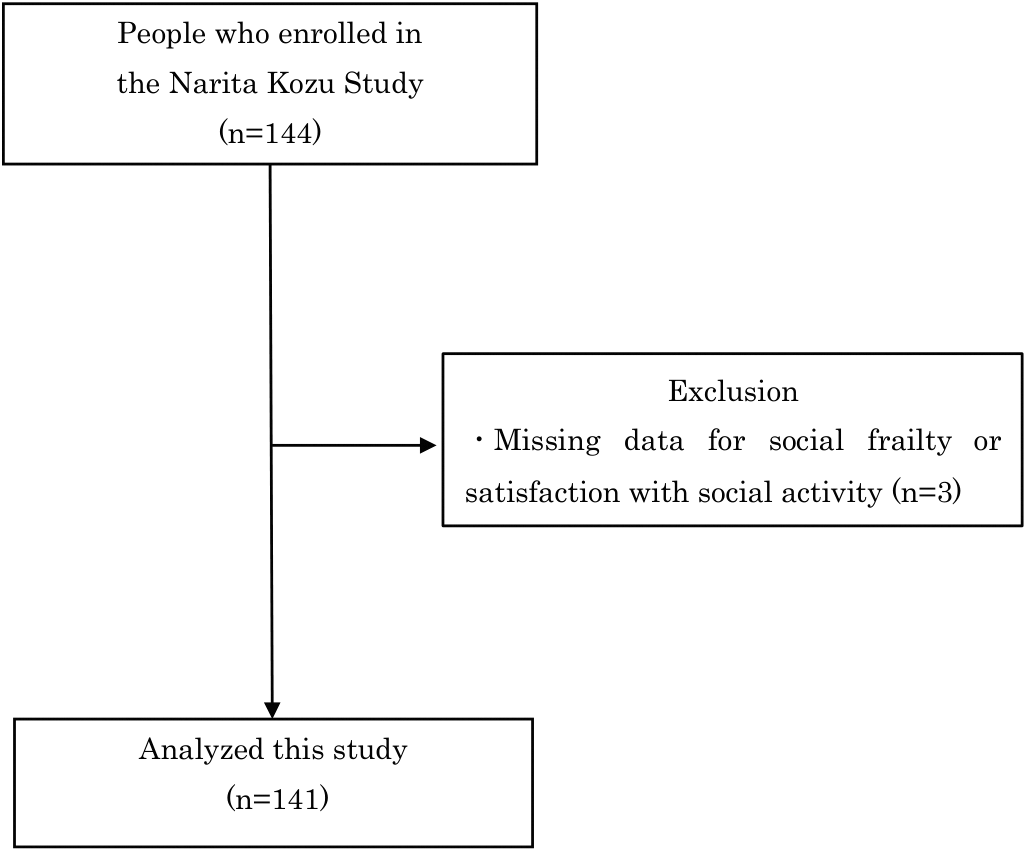
Flowchart for study inclusion and exclusion.

In total, 144 individuals participated in the health survey. After excluding three participants who met the exclusion criteria, 141 participants were included in the final analysis (Figure 1). Baseline characteristics are summarised in Table 1. Of the total sample, 103 participants (73.0%) were women, and the mean age ± standard deviation was 77.3 ± 5.8 years. Fifty participants (35.4%) were classified as having social frailty, 52 (36.9%) as having pre‐social frailty and 39 (27.7%) as robust. Detailed distributions of the MSFI item responses are shown in Table S1.

**TABLE 1:** Characteristics of the participants.

Variables	Overall (*n* = 141)	a. Social frail (*n* = 50)	b. Pre‐social frail (*n* = 52)	c. Robust (*n* = 39)	*p*	Post hoc comparison (*p*)
Demographic characteristics						
Age, year, mean ± SD	77.3 ± 5.8	78.0 ± 6.2	78.9 ± 5.1	74.2 ± 4.8	< 0.001^a^	a < c (0.003), b < c (< 0.001)^e^
Sex, Female (%)	103 (73.0)	39 (78.0)	39 (75.0)	25 (64.1)	0.315^c^	
Education, year, median (IQR)	12.0 (12.0–15.0)	12.0 (12.0–14.0)	12.0 (12.0–15.8)	14.0 (12.0–16.0)	0.321^b^	
Living arrangement, alone (%)	45 (31.9)	30 (60.0)	15 (28.8)	0 (0)	< 0.001^c^	a < b (0.002), b < c (< 0.001), a < c (< 0.001)^f^
Chronic diseases						
Hypertension, *n*, (%)	68 (48.2)	25 (50.0)	29 (55.8)	14 (35.9)	0.163^c^	
Diabetes, *n*, (%)	22 (15.6)	9 (18.0)	8 (15.4)	5 (12.8)	0.799^c^	
Heart disease, *n*, (%)	14 (9.9)	2 (4.0)	8 (15.4)	4 (10.3)	0.140^d^	
Stroke, *n*, (%)	3 (2.1)	0 (0.0)	1 (1.9)	2 (5.1)	0.276^d^	
Functional status						
Walking speed, m/s, median (IQR)	1.4 (1.2–1.5)	1.3 (1.2–1.5)	1.3 (1.2–1.5)	1.4 (1.3–1.5)	0.051^b^	
Cognitive function						
MoCA‐J, score, median (IQR)	25.0 (23.0–27.0)	25.0 (22.0–26.0)	25.0 (23.0–27.0)	26.0 (24.0–28.0)	0.052^b^	
Depression symptoms						
GDS‐15, score, median (IQR)	4.0 (1.0–6.0)	4.5 (2.0–8.0)	4.0 (1.3–6.0)	1.0 (1.0–4.0)	< 0.001^b^	a < c (< 0.001), b < c (0.003)^f^
ADL status						
Independence, *n*, (%)	141 (100)	50 (100)	52 (100)	39 (100)	N/A	
The SARDLSS, score, mean ± SD						
Total score	49.9 ± 10.7	45.0 ± 10.2	50.6 ± 11.3	55.3 ± 7.2	< 0.001^a^	a < b (0.014), a < c (< 0.001)^e^
Learning‐related activities	14.2 ± 3.6	13.0 ± 3.3	14.3 ± 4.1	15.6 ± 2.5	0.003^a^	a < c (0.002)^e^
Contributions to others and society	13.4 ± 3.7	11.8 ± 3.8	13.6 ± 3.7	15.1 ± 3.0	< 0.001^a^	a < b (0.028), a < c (< 0.001)^e^
Health and physical fitness	10.3 ± 2.6	9.3 ± 2.5	10.4 ± 2.7	11.5 ± 2.2	< 0.001^a^	a < c (< 0.001)^e^
Friendships	12.0 ± 2.7	10.8 ± 3.0	12.3 ± 2.5	13.2 ± 1.9	< 0.001^a^	a < b (0.014), a < c (< 0.001)^e^

Abbreviations: ADL, activities of daily living; GDS‐15, Geriatric Depression Scale‐15; IQR, interquartile range; MoCA‐J, Montreal Cognitive Assessment Japanese Version; N/A, not applicable; SARDLSS, Social Activities‐Related Daily Life Satisfaction Scale; SD, standard deviation.

^a^ One‐way ANOVA.

^b^ Kruskal–Wallis test.

^c^ Chi‐Square test.

^d^ Fisher's exact test.

^e^ Tukey's honestly significant difference.

^f^ Bonferroni correction (*p* < 0.017).

Between‐group comparisons by social frailty status are presented in Table 1. SARDLSS scores were significantly lower in the social frailty group than in both the pre‐social frailty and robust groups. GDS‐15 scores were significantly higher in both the social frailty and pre‐social frailty groups compared with the robust group. Participants in the social frailty and pre‐social frailty groups were significantly older than those in the robust group. Regarding family composition, the proportion of participants living alone increased significantly with increasing social frailty severity.

### 3.2 Association Between Social Frailty and Satisfaction With the Outcomes of Social Activities

Table 2 presents the results of ordinal logistic regression analysis assessing the association between social frailty status and satisfaction with the outcomes of social activities. In Model 1, after adjusting for age, sex and years of education, higher SARDLSS scores were significantly associated with lower odds of belonging to a more severe social frailty category (OR = 0.93, 95% CI: 0.90–0.97, *p* < 0.001). Age was also significantly associated with social frailty status, with participants aged 75 years or older being more likely to exhibit more severe social frailty (OR = 2.19, 95% CI: 1.09–4.42, *p* = 0.028).

**TABLE 2:** Ordinal logistic regression analysis for social frailty status.

Variables	Model 1		Model 2	
	OR (95% CI)	*p*	OR (95% CI)	*p*
SARDLSS	0.93 (0.90–0.97)	< 0.001	0.95 (0.91–0.98)	0.004
Age (≥ 75 years)	2.19 (1.09–4.42)	0.028	1.85 (0.87–3.93)	0.109
Sex (female)	1.52 (0.71–3.26)	0.278	1.81 (0.80–4.10)	0.158
Years of education	1.06 (0.89–1.26)	0.544	1.10 (0.91–1.32)	0.334
Walking speed			0.30 (0.06–1.37)	0.120
MoCA‐J			0.94 (0.83–1.06)	0.318
GDS‐15			1.20 (1.06–1.36)	0.003
Model fit				
Pearson goodness‐of‐fit		0.379		0.623
Deviance goodness‐of‐fit		0.164		0.598

*Note:* Model 1 was adjusted for demographic factors, including age, sex and years of education. Model 2 additionally included variables related to gait speed, cognitive function and depressive symptoms. The dependent variable was social frailty status (0 = robust, 1 = pre‐social frailty, 2 = social frailty). Values are presented as odds ratios (ORs) with 95% confidence intervals (CIs). The proportional odds assumption was satisfied for all models (test of parallel lines, *p* > 0.05).

Abbreviations: GDS‐15, Geriatric Depression Scale‐15; MoCA‐J, Montreal Cognitive Assessment Japanese Version; SARDLSS, Social Activities‐Related Daily Life Satisfaction Scale.

In Model 2, after additionally adjusting for walking speed, MoCA‐J scores, and GDS‐15 scores, satisfaction with the outcomes of social activities remained significantly associated with social frailty status (OR = 0.95, 95% CI: 0.91–0.98, *p* = 0.004). In this model, depressive symptoms as assessed by the GDS‐15 were also significantly associated with social frailty status (OR = 1.20, 95% CI: 1.06–1.36, *p* = 0.003). The proportional odds assumption was satisfied in all models, and no issues with model fit were identified.

As a secondary analysis, domain‐level ordinal logistic regression models were fitted to examine the associations between each SARDLSS domain and social frailty status (Table 3). After Bonferroni correction (*p* < 0.0125), satisfaction with contributions to others and society (OR = 0.88, 95% CI: 0.80–0.97), health and physical fitness (OR = 0.84, 95% CI: 0.73–0.96) and friendships (OR = 0.79, 95% CI: 0.68–0.91) showed significant associations with social frailty status. In contrast, satisfaction with learning‐related activities was not significantly associated with social frailty status (OR = 0.92, 95% CI: 0.83–1.03).

**TABLE 3:** Associations between each SARDLSS domain and social frailty status.

SARDLSS domains	OR (95% CI)	*p*
Learning‐related activities	0.92 (0.83–1.03)	0.138
Contributions to others and society	0.88 (0.80–0.97)	0.010
Health and physical fitness	0.84 (0.73–0.96)	0.010
Friendships	0.79 (0.68–0.91)	0.002

*Note:* Each domain was analysed using a separate ordinal logistic regression model adjusted for age, sex, years of education, gait speed, cognitive function and depressive symptoms. The dependent variable was social frailty status (0 = robust, 1 = pre‐social frailty, 2 = social frailty). Values are presented as odds ratios (ORs) with 95% confidence intervals (CIs). The proportional odds assumption was satisfied for all domain‐specific models (test of parallel lines, *p* > 0.05). Bonferroni correction was applied with statistical significance set at *p* < 0.0125.

Abbreviation: SARDLSS, Social Activities‐Related Daily Life Satisfaction Scale.

In the sensitivity analysis, the modified SARDLSS score excluding the ‘satisfaction with contributions to others and society’ subdomain remained significantly associated with social frailty status in both Model 1 (OR = 0.91, 95% CI: 0.87–0.95) and Model 2 (OR = 0.93, 95% CI: 0.88–0.98), consistent with the primary analysis. The full results are presented in Table S2.

## 4 Discussion

We examined the association between social frailty and satisfaction with the outcomes of social activities among community‐dwelling older adults. Ordinal logistic regression indicated that satisfaction with the outcomes of social activities was independently associated with social frailty, even after adjusting for age, sex, educational attainment, walking speed, cognitive function and depressive symptoms. These findings suggest that satisfaction with the outcomes of social activities may represent a distinct psychological dimension of social frailty beyond established correlates such as physical functional decline and depressive symptoms. Collectively, social frailty may involve a quantitative reduction in social engagement and a qualitative decline in subjective satisfaction with the outcomes of social activities. Notably, in this study, we extend previous research by specifically focusing on satisfaction with the outcomes of social activities, rather than general well‐being indicators, thereby providing a more nuanced understanding of the qualitative dimension of social participation.

The prevalence of social frailty observed in the present study exceeded estimates reported in prior studies. Prior research has placed the prevalence of social frailty at approximately 20% [4, 9]. This discrepancy may be attributable, at least in part, to the older mean age of the participants (77.3 ± 5.8 years) of this study, as social frailty has been shown to increase with advancing age.

The ordinal logistic regression analysis revealed a graded relationship between satisfaction and the severity of social frailty, where lower satisfaction was associated with a higher likelihood of being classified into more severe categories. This finding suggests a continuum rather than discrete group differences, indicating that subjective satisfaction derived from social activities may decline stepwise as individuals transition from robust to pre‐social frailty and from pre‐social frailty to social frailty.

Domain‐level analyses further revealed that satisfaction in specific areas—namely friendships, health and physical fitness and contributions to others and society—was significantly associated with social frailty. These findings suggest that social frailty is characterised by limitations in behavioural and environmental aspects of social participation and by reduced satisfaction with the outcomes derived from engagement in these domains. Furthermore, these results are consistent with previous research reporting that lower levels of Ikigai (sense of purpose in life) are associated with social frailty among older women [19]. Taken together, social frailty may be conceptualised as a condition marked by reduced social behaviour or disadvantaged social environments and as a state in which individuals are less able to derive sufficient meaning and fulfilment from social engagement.

As we used a cross‐sectional design, causal relationships could not be directly established. Therefore, the directionality of the observed association remains unclear; however, a bidirectional association between social frailty and satisfaction with the outcomes of social activities may also be possible. As social frailty is characterised by reduced social roles and interactions, subjective evaluations such as perceived usefulness and fulfilment may also be lower, which may be related to lower satisfaction with the outcomes of social activities. Indeed, previous studies have reported that social frailty is directly associated with life satisfaction [20] and that older adults who are more actively engaged in social activities tend to report higher subjective satisfaction [21, 22]. Conversely, diminished satisfaction or meaning derived from social activities may be associated with lower motivation for social participation and with reduced activity frequency and social interactions, which may in turn be related to more severe social frailty. In older adults, higher levels of Ikigai and life satisfaction have been shown to be associated with the maintenance of social participation [23, 24], suggesting that psychological factors play a crucial role in shaping social participation behaviours. Collectively, social frailty and satisfaction with the outcomes of social activities may be interrelated, and they potentially form reciprocal associations. Prospective longitudinal and interventional studies are warranted to examine the temporal relationships between changes in satisfaction with the outcomes of social activities and the progression of social frailty.

The findings of this study have important practical implications for community‐dwelling older adults. Previous research on preventive care has largely prioritised the quantitative aspects of social participation, such as participation frequency or the presence or absence of participation [25, 26]. However, the present study reported that the quantity and quality of social participation—specifically, satisfaction with the outcomes of social activities—are closely associated with social frailty. The graded association observed in this study indicates that satisfaction may decline progressively as social frailty worsens. Furthermore, domain‐level analyses identified friendships, health and physical fitness and contributions to others and society as particularly relevant domains. Taken together, these results suggest that interventions for social frailty should move beyond simply increasing opportunities for social participation. Greater emphasis should be placed on enhancing the subjective value and meaning derived from social activities.

This study has some limitations. First, because of its cross‐sectional design, a causal relationship between social frailty and satisfaction with the outcomes of social activities cannot be established. Consequently, the observed associations do not indicate directionality or temporal sequence, and the findings should be interpreted with caution. Second, the participants were limited to older adults who were independent in ADL, resulting in no variability in ADL status; therefore, ADL could not be included as a covariate in the regression analyses. As ADL capacity may influence both social frailty and satisfaction with the outcomes of social activities, its potential confounding effect cannot be excluded. The present findings should therefore be interpreted with caution, and future studies incorporating participants with a broader range of ADL capacity are warranted. In addition, the study was conducted among older adults residing in a single region of Japan, which may restrict the generalisability of the results to other regions or populations with different backgrounds. Furthermore, the mean SARDLSS score in this study exceeded that reported in the original scale development study [11]. This suggests that our sample may have included a greater proportion of older adults with higher satisfaction regarding their social activities. As participants were recruited via community‐based flyers, selection bias towards individuals with greater interest in social activities may have been introduced. Third, the scale used to assess satisfaction with the outcomes of social activities was developed within the Japanese cultural context; thus, its validity and reliability outside Japan remain to be established. Fourth, we did not fully account for factors such as economic status or access to social resources, and the potential influence of these unmeasured confounders cannot be excluded. Prospective longitudinal studies are needed to clarify the temporal relationship between satisfaction with the outcomes of social activities and social frailty, while incorporating a broader range of background factors. Fifth, some degree of item similarity exists between the MSFI and the SARDLSS. Although these instruments assess distinct constructs, such similarity may have partially influenced the observed associations. However, sensitivity analyses excluding content‐wise similar subdomains yielded consistent results, suggesting that the impact of this similarity is likely limited. However, future studies including measures with greater construct independence are warranted.

In conclusion, in this study, we examined the association between social frailty and satisfaction with the outcomes of social activities among community‐dwelling older adults. The results demonstrated that satisfaction with the outcomes of social activities was independently associated with social frailty even after adjusting for age, sex, educational attainment, walking speed, cognitive function and depressive symptoms. Domain‐level analyses further revealed that satisfaction related to friendships, health and physical fitness and contributions to others and society was particularly strongly associated with social frailty status. These findings suggest that social frailty is closely associated with quantitative aspects of social participation, such as participation frequency, and with qualitative dimensions, including the subjective satisfaction and meaning derived from social activities. Therefore, future community‐based interventions should be aimed at increasing opportunities for social participation and at highlighting the importance of supporting older adults in finding value and fulfilment in their social activities. Such a perspective may be useful for understanding the association with social frailty.

## Conflicts of Interest

The authors declare no conflicts of interest.

## Supporting information


**Table S1:** Distribution of MSFI item responses across social frailty categories.

**Table S2:** Sensitivity analysis: association between modified SARDLSS and social frailty status.

## Data Availability

Data supporting the findings of this study are available upon request from the corresponding author. Owing to privacy or ethical constraints, the data are not publicly available.
