# Supplementary material for: Association Between Social Frailty and Satisfaction With the Outcomes of Social Activities in Community‐Dwelling Older Adults in Japan: A Cross‐Sectional Study
Source: Psychogeriatrics. 2026 May 6;26:e70177. doi: 10.1111/psyg.70177 (PMC13148954; doi:10.1111/psyg.70177)
Supplement: Supplementary file 1 — Table S1: Distribution of MSFI item responses across social frailty categories. [file PSYG-26-0-s001.docx]

**Supplementary table 1. Distribution of MSFI item responses across social frailty categories**

|  | Overall  (n=141) | 1. Social frail   (n=50) | 1. Pre-social frail   (n=52) | (c) Robust  (n=39) | *p* | Post hoc comparison (*p*) |
| --- | --- | --- | --- | --- | --- | --- |
| The MSFI items, n, (%) | | | | | | |
| 1. decreased frequency of going out compared with the previous year (yes) | 48 (34.0) | 29 (58.0) | 19 (36.5) | 0 (0) | <0.001^‡^ | b>c (<0.001), a>c (<0.001) ^‖^ |
| 1. visiting friends’ homes (no) | 93 (66.0) | 39 (78.0) | 16 (30.8) | 0 (0) | <0.001^‡^ | a>b (<0.001), b>c (<0.001), a>c (<0.001) ^‖^ |
| 1. perceiving oneself as useful to family or friends (no) | 11 (7.8) | 10 (20.0) | 1 (1.9) | 0 (0) | <0.001^§^ | a>b (0.003), a>c (0.002) ^‖^ |
| 1. living alone (yes) | 45 (31.9) | 30 (60.0) | 15 (28.8) | 0 (0) | <0.001^‡^ | a>b (0.002), b>c (<0.001), a>c (<0.001) ^‖^ |
| 1. having daily conversations with someone (no) | 15 (10.6) | 14 (28.0) | 1 (1.9) | 0 (0) | <0.001^‡^ | a>b (<0.001), a>c (<0.001) ^‖^ |

^‡^Chi-Square test; ^§^Fisher’s exact test; ^‖^Bonferroni correction (p < 0.017)

Abbreviations: MSFI, Makizako Social Frailty Index
