# Supplementary material for: Association Between Social Frailty and Satisfaction With the Outcomes of Social Activities in Community‐Dwelling Older Adults in Japan: A Cross‐Sectional Study
Source: Psychogeriatrics. 2026 May 6;26:e70177. doi: 10.1111/psyg.70177 (PMC13148954; doi:10.1111/psyg.70177)
Supplement: Supplementary file 2 — Table S2: Sensitivity analysis: association between modified SARDLSS and social frailty status. [file PSYG-26-0-s002.docx]

**Supplementary table 2. Sensitivity analysis: association between modified SARDLSS and social frailty status**

| Variables | Model 1 | | Model 2 | |
| --- | --- | --- | --- | --- |
|  | OR (95% CI) | *p* | OR (95% CI) | *p* |
| modified SARDLSS  (excluding the “contributions to others and society” domain) | 0.91 (0.87-0.95) | <0.001 | 0.93 (0.88-0.98) | 0.006 |
| Age (≥75 years) | 2.29 (1.14-4.62) | 0.021 | 1.91 (0.90-4.04) | 0.092 |
| Sex (female) | 1.74 (0.80-3.75) | 0.161 | 1.99 (0.86-4.56) | 0.106 |
| Years of education | 1.06 (0.89-1.26) | 0.520 | 1.10 (0.91-1.32) | 0.330 |
| Walking speed |  |  | 0.31 (0.07-1.41) | 0.128 |
| MoCA-J |  |  | 0.94 (0.83-1.06) | 0.306 |
| GDS-15 |  |  | 1.20 (1.06-1.35) | 0.003 |
| Model fit |  |  |  |  |
| Pearson goodness-of-fit |  | 0.755 |  | 0.624 |
| Deviance goodness-of-fit |  | 0.391 |  | 0.588 |

Model 1 was adjusted for demographic factors, including age, sex, and years of education.

Model 2 additionally included variables related to gait speed, cognitive function, and depressive symptoms.

The dependent variable was social frailty status (0 = robust, 1 = pre-social frailty, 2 = social frailty).

Values are presented as odds ratios (ORs) with 95% confidence intervals (CIs).

The proportional odds assumption was satisfied for all models (test of parallel lines, p > 0.05).

Abbreviations: MoCA-J, Montreal Cognitive Assessment Japanese Version; GDS-15, Geriatric Depression Scale-15; SARDLSS,

Social Activities-Related Daily Life Satisfaction Scale
